# Bis[*cis*-bis­(diphenyl­phosphino)ethene]copper(I) dichloridocuprate(I)

**DOI:** 10.1107/S1600536810012146

**Published:** 2010-04-10

**Authors:** Peter C. Healy, John C. McMurtrie, Jocelyne Bouzaid

**Affiliations:** a Eskitis Institute for Cell and Molecular Therapies, Griffith University, Brisbane 4111, Australia; b Chemistry, Faculty of Science and Technology, Queensland University of Technology, Brisbane, 4001, Australia

## Abstract

The crystal structure of the title compound, [Cu(C_26_H_22_P_2_)_2_][CuCl_2_], is composed of discrete Cu(dppey)_2_]^+^ cations [dppey is *cis*-bis­(diphenyl­phosphino)ethene] and [CuCl_2_]^−^ anions. The tetra­hedral Cu(P—P)_2_ core of the [Cu(dppey)_2_]^+^ cation is distorted, with Cu—P bond lengths ranging from 2.269 (1) to 2.366 (1) Å. The five-membered –Cu—P—CH=CH—P– rings adopt envelope conformations, with the Cu atom lying 0.38 and 0.65 Å out of the P—C=C—P planes. The Cu—Cl distances in the [CuCl_2_]^−^ anion are 2.094 (2) and 2.096 (2) Å, with a Cl—Cu—Cl angle of 176.81 (7)°.

## Related literature

For related literature and crystal structures of [Cu(dppey)_2_]^+^ complexes, see: Berners-Price *et al.* (1992 ▶); Healy *et al.* (2009 ▶). For background literature and crystal structures of [CuCl_2_]^−^ complexes, see: Rodenstein *et al.* (2008 ▶); Wang *et al.* (2005 ▶); Mirkhani *et al.* (2004 ▶); Healy *et al.* (1989 ▶); Asplund *et al.* (1983 ▶). For Raman spectroscopy of [CuCl_2_]^−^ complexes, see: Bowmaker *et al.* (1973 ▶, 2007 ▶). For distortion parameters in tetra­hedral bidentate complexes, see: Dobson *et al.* (1984 ▶); Healy *et al.* (2008 ▶).
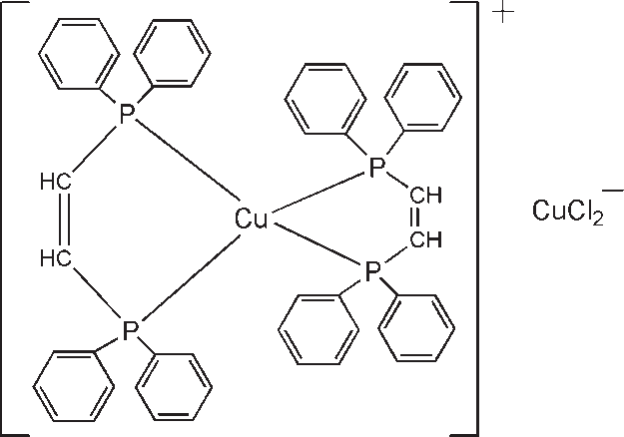


## Experimental

### 

#### Crystal data

[Cu(C_26_H_22_P_2_)_2_][CuCl_2_]
*M* *_r_* = 990.75
Monoclinic, 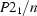

*a* = 15.3109 (9) Å
*b* = 16.1519 (11) Å
*c* = 18.6419 (8) Å
β = 95.950 (4)°
*V* = 4585.3 (5) Å^3^
*Z* = 4
Mo *K*α radiation
μ = 1.22 mm^−1^
*T* = 223 K
0.45 × 0.34 × 0.32 mm

#### Data collection

Oxford Diffraction GEMINI S Ultra diffractometer
Absorption correction: multi-scan (*CrysAlis PRO*; Oxford Diffraction, 2009 ▶) *T*_min_ = 0.610, *T*_max_ = 0.696
18820 measured reflections
8015 independent reflections
6315 reflections with *I* > 2σ(*I*)
*R*_int_ = 0.038

#### Refinement

*R*[*F* ^2^ > 2σ(*F* ^2^)] = 0.052
*wR*(*F* ^2^) = 0.140
*S* = 1.10
8015 reflections
541 parameters
H-atom parameters constrained
Δρ_max_ = 2.43 e Å^−3^
Δρ_min_ = −1.04 e Å^−3^

### 

Data collection: *CrysAlis CCD* (Oxford Diffraction, 2009 ▶); cell refinement: *CrysAlis RED* (Oxford Diffraction, 2009 ▶); data reduction: *CrysAlis RED*; program(s) used to solve structure: *SIR97* (Altomare *et al.*, 1999 ▶); program(s) used to refine structure: *SHELXL97* (Sheldrick, 2008 ▶); molecular graphics: *ORTEP-3 for Windows* (Farrugia, 1997 ▶); software used to prepare material for publication: *PLATON* (Spek, 2009 ▶).

## Supplementary Material

Crystal structure: contains datablocks global, I. DOI: 10.1107/S1600536810012146/nk2029sup1.cif

Structure factors: contains datablocks I. DOI: 10.1107/S1600536810012146/nk2029Isup2.hkl

Additional supplementary materials:  crystallographic information; 3D view; checkCIF report

## Figures and Tables

**Fig. 1.**
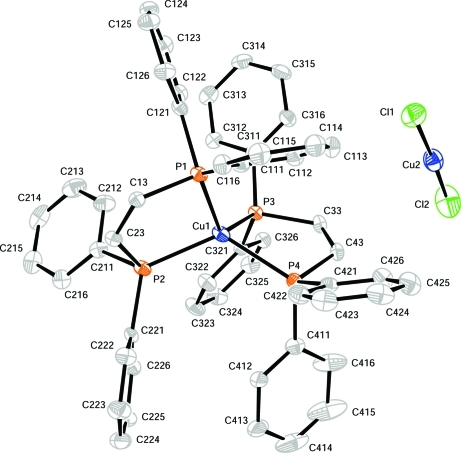
View of the cation and anion of the title complex. Displacement ellipsoids for non-H atoms are drawn at the 30% probability level. H atoms are omitted for clarity.

## supplementary crystallographic information

### Comment

Previous single crystal structure determinations on the 1:2 adducts of copper(I) salts with the bidentate phosphine ligand, Ph_2_P(CH=CH)PPh_2_ (dppey), show the formation of stable bis-chelated ionic complexes [Cu(dppey)_2_]X for X = PF_6_ (Berners-Price *et al.*, 1992) and for BF_4_ as an ethanol solvate (Healy *et al.*, 2009). In this present work, addition of aqueous hydrochloric acid to a suspension of copper(I) oxide in a solution of dppey in ethanol resulted in the dissolution of the red copper oxide and subsequent precipitation of crystals of the title complex, [Cu(dppey)_2_][CuCl_2_] (I), the structure of which is reported here.

The crystal structure consists of discrete Cu(dppey)_2_]^+^ cations and [CuCl_2_]^-^ anions (Fig. 1). In the cation, the four Cu—P bond lengths are dispersed over the range 2.269 (1) - 2.366 (1) Å. The overall Cu(P—P)_2_ coordination geometry about the copper atom is distorted tetrahedral with the intra-ligand 'bite' angles 89.61 (4) and 87.15 (4)° while the the P—Cu—P inter-ligand angles range from 115.54 (4) - 123.27 (4)°. Angular distortion of the Cu(L—L)_2_ core of four-coordinate bis(bidentate) complexes can be conveniently described by the angular distortion parameters θ_x_, θ_y_ and θ_z_, where θ_x_ and θ_y_ represent rocking motions of the two CuP_2_ planes with respect to each other and θ_z_ the degree of twist between the two planes (Dobson *et al.*, 1984; Healy *et al.*, 2008). For complexes with D_2 d_ symmetry, θ_x_ = θ_y_ = θ_z_ = 90°. For this present cation, the values of θ_x_, θ_y_ and θ_z_, are 93.4, 86.9 and 92.8°. The five membered -Cu—P—CH=CH—P- rings adopt envelope conformations with the copper atom lying 0.38Å out of the P1—C12=C23—P2 plane and 0.65Å out of the P3—C33=C43—P4 plane.

These results show significant differences from those observed for both the PF_6_ and BF_4_ complexes, in which the Cu—P bond lengths span narrow ranges of 2.276 (2)-2.289 (2)Å and 2.272 (1)-2.282 (1)Å respectively. The parameters θ_x_, θ_y_ and θ_z_ are 90.4, 90.4 and 108.6° for the PF_6_ complex and 90.5, 89.7 and 72.7° for the BF_4_ complex; while the distances of the copper from the ligand planes are 0.03, 0.21Å and 0.04,0.21Å respectively.

The Cu—Cl distances in the anion are 2.094 (2) and 2.096 (2) Å. The anion deviates from linearity with the Cl—Cu—Cl angle 176.81 (7)°. These values are in accord with those reported for other compounds incorporating the [CuCl_2_]^-^ anion (e.g. Rodenstein *et al.*, 2008; Wang *et al.*, 2005: Mirkhani *et al.*, 2004; Healy *et al.*, 1989; Asplund *et al.*, 1983). Four C—H···Cl contacts distances ranging between 2.9 and 3.0Å are observed in the structure (Cl1···H314^i^ 2.88 Å, Cl1···H323^ii^ 2.99 Å, Cl2···H115^iii^ 3.03 Å, Cl2···H33 3.00 Å; symmetry codes: (i) 1-x, -y, 2-z, (ii) x-1/2, 1/2-y, 1/2+z, (iii) x-1/2, 1/2-y, z-1/2).

Both the symmetric and anti-symmetic Cu—Cl stretching modes would be expected to be Raman active in this non linear (C_2v_) anion and in the solid state Raman spectrum of this complex we have assigned two bands of equal intensity observed at 304 and 319 cm^-1^ not present in the spectrum of the free ligand to the ν(Cu—Cl) stretching modes (cf. Bowmaker *et al.*, 1973; 2007).

### Experimental

A concentrated aqueous solution of HCl was added dropwise to a suspension of Cu_2_O (0.067 g, 0.47 mmol) in a stirred solution of dppey (0.309 g, 0.78 mmol) in 10 ml e thanol until all the Cu_2_O dissolved and a white precipitate formed. The volume of the reaction mixture was increased to 30 ml s and heated to reflux to give a clear solution. This was allowed to slowly cool to room temperature to give colourless crystals of the title complex suitable for single crystal X-ray diffraction studies. M.p. 490-491 K. Raman spectra on for the complex and ligand were recorded on a Renishaw InVia spectrometer.

### Refinement

H atoms attached to carbons were constrained as riding atoms, with C–H set to 0.95 Å. U_iso_(H) values were set to 1.2U_eq_ of the parent atom. Maximum residual electron density in the complex was located at 0.9Å from the cationic copper site.

### Crystal data

**Table:**

[Cu(C_26_H_22_P_2_)_2_][CuCl_2_]	*F*(000) = 2032
*M**_r_* = 990.75	*D*_x_ = 1.435 Mg m^−^^3^
Monoclinic, *P*2_1_/*n*	Mo *K*α radiation, λ = 0.71070 Å
Hall symbol: -P 2yn	Cell parameters from 8706 reflections
*a* = 15.3109 (9) Å	θ = 3.2–32.5°
*b* = 16.1519 (11) Å	µ = 1.22 mm^−^^1^
*c* = 18.6419 (8) Å	*T* = 223 K
β = 95.950 (4)°	Block, colourless
*V* = 4585.3 (5) Å^3^	0.45 × 0.34 × 0.32 mm
*Z* = 4	

### Data collection

**Table:**

Oxford Diffraction GEMINI S Ultra diffractometer	8015 independent reflections
Radiation source: Enhance (Mo) X-ray Source	6315 reflections with *I* > 2σ(*I*)
graphite	*R*_int_ = 0.038
Detector resolution: 16.0774 pixels mm^-1^	θ_max_ = 25.0°, θ_min_ = 3.3°
ω and φ scans	*h* = −18→17
Absorption correction: multi-scan (*CrysAlis PRO*; Oxford Diffraction, 2009)	*k* = −17→19
*T*_min_ = 0.610, *T*_max_ = 0.696	*l* = −14→22
18820 measured reflections	

### Refinement

**Table:**

Refinement on *F*^2^	Primary atom site location: structure-invariant direct methods
Least-squares matrix: full	Secondary atom site location: difference Fourier map
*R*[*F*^2^ > 2σ(*F*^2^)] = 0.052	Hydrogen site location: inferred from neighbouring sites
*wR*(*F*^2^) = 0.140	H-atom parameters constrained
*S* = 1.10	*w* = 1/[σ^2^(*F*_o_^2^) + (0.0716*P*)^2^ + 4.4671*P*] where *P* = (*F*_o_^2^ + 2*F*_c_^2^)/3
8015 reflections	(Δ/σ)_max_ = 0.001
541 parameters	Δρ_max_ = 2.43 e Å^−^^3^
0 restraints	Δρ_min_ = −1.04 e Å^−^^3^

### Special details

**Table:**

Geometry. Bond distances, angles etc. have been calculated using the rounded fractional coordinates. All su's are estimated from the variances of the (full) variance-covariance matrix. The cell esds are taken into account in the estimation of distances, angles and torsion angles
Refinement. Refinement on *F*^2^ for ALL reflections except those flagged by the user for potential systematic errors. Weighted *R*-factors wR and all goodnesses of fit *S* are based on *F*^2^, conventional *R*-factors *R* are based on *F*, with *F* set to zero for negative *F*^2^. The observed criterion of *F*^2^ > σ(*F*^2^) is used only for calculating -*R*-factor-obs etc. and is not relevant to the choice of reflections for refinement. *R*-factors based on *F*^2^ are statistically about twice as large as those based on *F*, and *R*-factors based on ALL data will be even larger.

### Fractional atomic coordinates and isotropic or equivalent isotropic displacement parameters (Å^2^)

**Table:**

	*x*	*y*	*z*	*U*_iso_*/*U*_eq_	
Cu1	0.80116 (3)	0.21062 (3)	0.95590 (2)	0.0312 (1)	
P1	0.82828 (6)	0.11348 (6)	1.04550 (5)	0.0277 (3)	
P2	0.93393 (6)	0.18014 (6)	0.91540 (5)	0.0290 (3)	
P3	0.67319 (6)	0.21508 (6)	0.88279 (4)	0.0253 (3)	
P4	0.76677 (6)	0.34498 (6)	0.99506 (5)	0.0300 (3)	
C13	0.9445 (2)	0.0902 (2)	1.04169 (18)	0.0267 (10)	
C23	0.9876 (2)	0.1169 (2)	0.98804 (18)	0.0285 (11)	
C33	0.6106 (2)	0.2954 (2)	0.92250 (19)	0.0320 (11)	
C43	0.6491 (2)	0.3492 (2)	0.96959 (19)	0.0310 (11)	
C111	0.8199 (2)	0.1441 (2)	1.13855 (18)	0.0288 (11)	
C112	0.7422 (3)	0.1827 (3)	1.1532 (2)	0.0372 (12)	
C113	0.7297 (3)	0.2054 (3)	1.2234 (2)	0.0459 (16)	
C114	0.7943 (3)	0.1898 (3)	1.2786 (2)	0.0484 (16)	
C115	0.8713 (3)	0.1519 (3)	1.2650 (2)	0.0431 (15)	
C116	0.8849 (3)	0.1299 (3)	1.1949 (2)	0.0357 (11)	
C121	0.7829 (2)	0.0077 (2)	1.04322 (18)	0.0297 (11)	
C122	0.7268 (3)	−0.0186 (3)	0.98437 (19)	0.0341 (11)	
C123	0.6942 (3)	−0.0989 (3)	0.9820 (2)	0.0401 (14)	
C124	0.7168 (3)	−0.1530 (3)	1.0376 (2)	0.0425 (12)	
C125	0.7727 (3)	−0.1278 (3)	1.0972 (2)	0.0450 (16)	
C126	0.8049 (3)	−0.0473 (3)	1.10009 (19)	0.0366 (11)	
C211	0.9352 (3)	0.1096 (2)	0.83815 (19)	0.0332 (11)	
C212	0.8692 (3)	0.0504 (3)	0.8293 (2)	0.0490 (16)	
C213	0.8656 (4)	−0.0057 (3)	0.7733 (3)	0.0614 (19)	
C214	0.9258 (4)	−0.0020 (3)	0.7239 (3)	0.0574 (19)	
C215	0.9916 (3)	0.0562 (3)	0.7305 (2)	0.0534 (16)	
C216	0.9971 (3)	0.1125 (3)	0.7880 (2)	0.0407 (14)	
C221	1.0158 (2)	0.2587 (2)	0.90291 (18)	0.0270 (11)	
C222	1.0773 (3)	0.2805 (3)	0.95950 (19)	0.0384 (11)	
C223	1.1383 (3)	0.3432 (3)	0.9517 (2)	0.0495 (16)	
C224	1.1353 (3)	0.3864 (3)	0.8875 (3)	0.0503 (17)	
C225	1.0736 (3)	0.3667 (3)	0.8314 (2)	0.0443 (16)	
C226	1.0135 (3)	0.3031 (2)	0.8384 (2)	0.0356 (12)	
C311	0.5976 (2)	0.1280 (2)	0.87184 (18)	0.0286 (11)	
C312	0.6123 (3)	0.0674 (2)	0.8210 (2)	0.0345 (11)	
C313	0.5628 (3)	−0.0042 (3)	0.8162 (2)	0.0447 (14)	
C314	0.4979 (3)	−0.0164 (3)	0.8612 (3)	0.0487 (16)	
C315	0.4825 (3)	0.0421 (3)	0.9106 (3)	0.0505 (16)	
C316	0.5325 (3)	0.1148 (3)	0.9170 (2)	0.0418 (14)	
C321	0.6762 (2)	0.2503 (2)	0.78973 (18)	0.0268 (10)	
C322	0.7560 (3)	0.2637 (3)	0.7634 (2)	0.0402 (14)	
C323	0.7605 (3)	0.2870 (3)	0.6920 (2)	0.0503 (16)	
C324	0.6835 (3)	0.2970 (3)	0.6470 (2)	0.0463 (15)	
C325	0.6033 (3)	0.2851 (3)	0.6725 (2)	0.0430 (14)	
C326	0.5991 (3)	0.2620 (2)	0.74381 (19)	0.0328 (11)	
C411	0.8069 (3)	0.4333 (3)	0.9471 (2)	0.0408 (13)	
C412	0.8965 (4)	0.4409 (3)	0.9429 (4)	0.079 (2)	
C413	0.9290 (6)	0.5065 (4)	0.9057 (5)	0.109 (3)	
C414	0.8736 (7)	0.5646 (4)	0.8735 (3)	0.099 (3)	
C415	0.7853 (6)	0.5584 (4)	0.8783 (3)	0.101 (3)	
C416	0.7511 (4)	0.4939 (4)	0.9159 (3)	0.076 (2)	
C421	0.7838 (3)	0.3777 (2)	1.08940 (19)	0.0311 (11)	
C422	0.8642 (3)	0.3600 (3)	1.1275 (2)	0.0426 (14)	
C423	0.8832 (3)	0.3860 (3)	1.1984 (2)	0.0500 (16)	
C424	0.8211 (3)	0.4268 (3)	1.2323 (2)	0.0533 (16)	
C425	0.7404 (3)	0.4450 (3)	1.1957 (2)	0.0535 (16)	
C426	0.7216 (3)	0.4196 (3)	1.1241 (2)	0.0445 (14)	
Cu2	0.44649 (4)	0.31970 (4)	1.04879 (3)	0.0542 (2)	
Cl1	0.49493 (10)	0.23942 (9)	1.13188 (8)	0.0720 (5)	
Cl2	0.40010 (12)	0.39502 (13)	0.96164 (8)	0.0965 (7)	
H13	0.97490	0.05830	1.07910	0.0320*	
H23	1.04770	0.10270	0.98750	0.0340*	
H33	0.54900	0.29900	0.90940	0.0380*	
H43	0.61490	0.39030	0.99010	0.0370*	
H112	0.69760	0.19390	1.11510	0.0440*	
H113	0.67650	0.23130	1.23310	0.0540*	
H114	0.78580	0.20560	1.32650	0.0580*	
H115	0.91520	0.14060	1.30360	0.0510*	
H116	0.93890	0.10520	1.18550	0.0430*	
H122	0.71050	0.01830	0.94570	0.0410*	
H123	0.65610	−0.11650	0.94150	0.0480*	
H124	0.69410	−0.20790	1.03550	0.0500*	
H125	0.78870	−0.16530	1.13560	0.0530*	
H126	0.84220	−0.02960	1.14110	0.0430*	
H212	0.82600	0.04830	0.86240	0.0580*	
H213	0.82100	−0.04700	0.76890	0.0730*	
H214	0.92210	−0.04010	0.68480	0.0680*	
H215	1.03320	0.05810	0.69610	0.0640*	
H216	1.04290	0.15250	0.79300	0.0480*	
H222	1.07810	0.25230	1.00430	0.0460*	
H223	1.18140	0.35620	0.99040	0.0590*	
H224	1.17620	0.42970	0.88220	0.0590*	
H225	1.07180	0.39670	0.78740	0.0530*	
H226	0.97100	0.29010	0.79920	0.0420*	
H312	0.65650	0.07540	0.78960	0.0410*	
H313	0.57360	−0.04530	0.78170	0.0530*	
H314	0.46390	−0.06580	0.85760	0.0570*	
H315	0.43730	0.03380	0.94110	0.0600*	
H316	0.52180	0.15490	0.95230	0.0490*	
H322	0.80870	0.25700	0.79450	0.0480*	
H323	0.81580	0.29580	0.67440	0.0590*	
H324	0.68620	0.31250	0.59810	0.0560*	
H325	0.55080	0.29230	0.64120	0.0510*	
H326	0.54370	0.25420	0.76150	0.0390*	
H412	0.93590	0.40110	0.96570	0.0940*	
H413	0.99050	0.51090	0.90270	0.1300*	
H414	0.89650	0.60900	0.84780	0.1170*	
H415	0.74700	0.59890	0.85540	0.1210*	
H416	0.68990	0.49150	0.92050	0.0910*	
H422	0.90680	0.32970	1.10470	0.0510*	
H423	0.93940	0.37520	1.22330	0.0600*	
H424	0.83340	0.44280	1.28140	0.0630*	
H425	0.69790	0.47450	1.21910	0.0640*	
H426	0.66560	0.43130	1.09920	0.0530*	

### Atomic displacement parameters (Å^2^)

**Table:**

	*U*^11^	*U*^22^	*U*^33^	*U*^12^	*U*^13^	*U*^23^
Cu1	0.0253 (2)	0.0421 (3)	0.0255 (2)	−0.0024 (2)	−0.0001 (2)	0.0085 (2)
P1	0.0270 (5)	0.0350 (5)	0.0208 (4)	−0.0012 (4)	0.0017 (4)	0.0063 (4)
P2	0.0266 (5)	0.0378 (6)	0.0222 (4)	−0.0030 (4)	0.0011 (4)	0.0077 (4)
P3	0.0252 (5)	0.0304 (5)	0.0198 (4)	−0.0006 (4)	0.0001 (3)	0.0010 (4)
P4	0.0292 (5)	0.0353 (5)	0.0256 (5)	−0.0015 (4)	0.0036 (4)	0.0015 (4)
C13	0.0292 (19)	0.0286 (19)	0.0217 (16)	0.0023 (15)	0.0004 (14)	0.0062 (14)
C23	0.0245 (18)	0.034 (2)	0.0260 (18)	−0.0026 (15)	−0.0021 (14)	0.0038 (15)
C33	0.0266 (19)	0.042 (2)	0.0277 (18)	0.0069 (17)	0.0038 (15)	−0.0005 (17)
C43	0.0304 (19)	0.036 (2)	0.0273 (18)	0.0080 (17)	0.0060 (15)	0.0006 (16)
C111	0.036 (2)	0.0268 (19)	0.0243 (17)	−0.0041 (16)	0.0064 (15)	0.0049 (15)
C112	0.036 (2)	0.036 (2)	0.040 (2)	0.0012 (17)	0.0057 (17)	0.0040 (18)
C113	0.050 (3)	0.039 (2)	0.052 (3)	0.007 (2)	0.021 (2)	−0.005 (2)
C114	0.067 (3)	0.047 (3)	0.034 (2)	−0.006 (2)	0.018 (2)	−0.0094 (19)
C115	0.053 (3)	0.048 (3)	0.0278 (19)	−0.003 (2)	0.0014 (18)	−0.0018 (18)
C116	0.038 (2)	0.040 (2)	0.0288 (19)	0.0011 (18)	0.0025 (16)	−0.0017 (17)
C121	0.0280 (19)	0.038 (2)	0.0242 (17)	−0.0008 (16)	0.0074 (15)	0.0026 (15)
C122	0.035 (2)	0.041 (2)	0.0261 (18)	−0.0004 (17)	0.0026 (16)	−0.0014 (16)
C123	0.042 (2)	0.044 (3)	0.034 (2)	−0.0031 (19)	0.0026 (17)	−0.0109 (19)
C124	0.047 (2)	0.035 (2)	0.047 (2)	−0.0069 (19)	0.012 (2)	−0.0041 (19)
C125	0.056 (3)	0.040 (3)	0.039 (2)	−0.003 (2)	0.005 (2)	0.0096 (19)
C126	0.045 (2)	0.040 (2)	0.0239 (18)	−0.0067 (19)	−0.0010 (16)	0.0044 (16)
C211	0.039 (2)	0.036 (2)	0.0231 (17)	−0.0003 (17)	−0.0040 (15)	0.0058 (16)
C212	0.054 (3)	0.048 (3)	0.044 (2)	−0.013 (2)	0.001 (2)	0.000 (2)
C213	0.068 (4)	0.046 (3)	0.066 (3)	−0.011 (2)	−0.013 (3)	0.002 (3)
C214	0.074 (4)	0.047 (3)	0.045 (3)	0.016 (3)	−0.024 (3)	−0.012 (2)
C215	0.060 (3)	0.061 (3)	0.038 (2)	0.015 (3)	−0.001 (2)	−0.003 (2)
C216	0.042 (2)	0.047 (3)	0.032 (2)	0.0003 (19)	−0.0020 (17)	−0.0035 (19)
C221	0.0233 (18)	0.032 (2)	0.0271 (17)	0.0001 (15)	0.0087 (14)	−0.0006 (15)
C222	0.045 (2)	0.045 (2)	0.0249 (18)	−0.008 (2)	0.0028 (16)	−0.0016 (17)
C223	0.050 (3)	0.053 (3)	0.045 (2)	−0.018 (2)	0.002 (2)	−0.013 (2)
C224	0.053 (3)	0.042 (3)	0.058 (3)	−0.012 (2)	0.016 (2)	−0.002 (2)
C225	0.048 (3)	0.041 (3)	0.046 (2)	−0.003 (2)	0.015 (2)	0.014 (2)
C226	0.034 (2)	0.039 (2)	0.034 (2)	0.0011 (17)	0.0039 (16)	0.0058 (17)
C311	0.0252 (18)	0.035 (2)	0.0243 (17)	−0.0004 (15)	−0.0036 (14)	0.0066 (15)
C312	0.037 (2)	0.036 (2)	0.0292 (19)	0.0002 (17)	−0.0026 (16)	0.0002 (16)
C313	0.050 (3)	0.039 (2)	0.042 (2)	0.000 (2)	−0.010 (2)	−0.0048 (19)
C314	0.048 (3)	0.033 (2)	0.063 (3)	−0.010 (2)	−0.004 (2)	0.009 (2)
C315	0.038 (2)	0.053 (3)	0.062 (3)	−0.008 (2)	0.013 (2)	0.016 (2)
C316	0.038 (2)	0.046 (3)	0.043 (2)	0.0005 (19)	0.0121 (18)	0.003 (2)
C321	0.0311 (19)	0.0268 (19)	0.0220 (16)	0.0000 (15)	0.0010 (14)	0.0025 (14)
C322	0.034 (2)	0.055 (3)	0.031 (2)	0.0004 (19)	0.0003 (17)	0.0122 (19)
C323	0.047 (3)	0.067 (3)	0.039 (2)	−0.004 (2)	0.015 (2)	0.015 (2)
C324	0.058 (3)	0.055 (3)	0.0254 (19)	−0.009 (2)	0.0024 (19)	0.0089 (19)
C325	0.046 (2)	0.048 (3)	0.032 (2)	0.000 (2)	−0.0095 (18)	0.0062 (19)
C326	0.033 (2)	0.037 (2)	0.0276 (18)	0.0015 (17)	−0.0008 (15)	0.0027 (16)
C411	0.062 (3)	0.039 (2)	0.0234 (18)	−0.010 (2)	0.0139 (18)	−0.0039 (17)
C412	0.087 (4)	0.033 (3)	0.129 (5)	−0.005 (3)	0.076 (4)	−0.001 (3)
C413	0.150 (7)	0.037 (3)	0.163 (7)	−0.020 (4)	0.127 (6)	−0.014 (4)
C414	0.207 (9)	0.053 (4)	0.046 (3)	−0.054 (5)	0.055 (4)	−0.015 (3)
C415	0.168 (8)	0.067 (4)	0.056 (4)	−0.048 (5)	−0.045 (4)	0.027 (3)
C416	0.091 (4)	0.064 (4)	0.063 (3)	−0.034 (3)	−0.035 (3)	0.030 (3)
C421	0.038 (2)	0.031 (2)	0.0247 (18)	−0.0097 (16)	0.0048 (16)	−0.0001 (15)
C422	0.041 (2)	0.049 (3)	0.037 (2)	−0.010 (2)	0.0005 (18)	0.0002 (19)
C423	0.050 (3)	0.060 (3)	0.037 (2)	−0.013 (2)	−0.010 (2)	0.007 (2)
C424	0.066 (3)	0.066 (3)	0.027 (2)	−0.025 (3)	0.000 (2)	−0.002 (2)
C425	0.062 (3)	0.065 (3)	0.035 (2)	−0.009 (2)	0.012 (2)	−0.011 (2)
C426	0.044 (2)	0.055 (3)	0.034 (2)	−0.002 (2)	0.0011 (18)	−0.003 (2)
Cu2	0.0453 (3)	0.0650 (4)	0.0523 (3)	0.0000 (3)	0.0049 (3)	−0.0095 (3)
Cl1	0.0788 (10)	0.0607 (8)	0.0766 (9)	0.0045 (7)	0.0087 (7)	0.0063 (7)
Cl2	0.0892 (12)	0.1363 (16)	0.0624 (9)	0.0361 (11)	−0.0003 (8)	0.0138 (10)

### Geometric parameters (Å, °)

**Table:**

Cu1—P1	2.2979 (11)	C125—H125	0.9500
Cu1—P2	2.2941 (10)	C126—H126	0.9500
Cu1—P3	2.2689 (10)	C311—C312	1.397 (5)
Cu1—P4	2.3663 (11)	C311—C316	1.386 (5)
Cu2—Cl2	2.0940 (18)	C212—H212	0.9500
Cu2—Cl1	2.0958 (16)	C312—C313	1.381 (6)
P1—C111	1.821 (3)	C313—C314	1.379 (7)
P1—C13	1.827 (3)	C213—H213	0.9500
P1—C121	1.843 (3)	C314—C315	1.357 (7)
P2—C211	1.838 (4)	C214—H214	0.9500
P2—C23	1.823 (3)	C315—C316	1.400 (7)
P2—C221	1.816 (3)	C215—H215	0.9500
P3—C311	1.820 (3)	C216—H216	0.9500
P3—C321	1.831 (3)	C321—C326	1.397 (5)
P3—C33	1.816 (3)	C321—C322	1.380 (5)
P4—C421	1.829 (4)	C222—H222	0.9500
P4—C43	1.816 (3)	C322—C323	1.392 (5)
P4—C411	1.824 (5)	C223—H223	0.9500
C13—C23	1.326 (5)	C323—C324	1.384 (6)
C33—C43	1.329 (5)	C224—H224	0.9500
C111—C112	1.395 (6)	C324—C325	1.375 (6)
C111—C116	1.389 (5)	C225—H225	0.9500
C112—C113	1.391 (5)	C325—C326	1.389 (5)
C113—C114	1.375 (6)	C226—H226	0.9500
C13—H13	0.9500	C411—C412	1.388 (8)
C114—C115	1.375 (7)	C411—C416	1.387 (8)
C115—C116	1.391 (5)	C312—H312	0.9500
C121—C122	1.388 (5)	C412—C413	1.387 (10)
C121—C126	1.397 (5)	C313—H313	0.9500
C122—C123	1.389 (7)	C413—C414	1.362 (11)
C123—C124	1.372 (6)	C314—H314	0.9500
C23—H23	0.9500	C414—C415	1.368 (14)
C124—C125	1.391 (6)	C315—H315	0.9500
C125—C126	1.390 (7)	C415—C416	1.388 (9)
C33—H33	0.9500	C316—H316	0.9500
C43—H43	0.9500	C421—C426	1.382 (6)
C211—C212	1.389 (6)	C421—C422	1.386 (6)
C211—C216	1.399 (6)	C322—H322	0.9500
C212—C213	1.379 (7)	C422—C423	1.389 (5)
C112—H112	0.9500	C423—C424	1.365 (6)
C213—C214	1.370 (8)	C323—H323	0.9500
C113—H113	0.9500	C324—H324	0.9500
C114—H114	0.9500	C424—C425	1.380 (6)
C214—C215	1.374 (7)	C325—H325	0.9500
C115—H115	0.9500	C425—C426	1.398 (5)
C215—C216	1.402 (6)	C326—H326	0.9500
C116—H116	0.9500	C412—H412	0.9500
C221—C226	1.398 (5)	C413—H413	0.9500
C221—C222	1.385 (5)	C414—H414	0.9500
C122—H122	0.9500	C415—H415	0.9500
C222—C223	1.395 (7)	C416—H416	0.9500
C123—H123	0.9500	C422—H422	0.9500
C223—C224	1.382 (7)	C423—H423	0.9500
C124—H124	0.9500	C424—H424	0.9500
C224—C225	1.372 (7)	C425—H425	0.9500
C225—C226	1.394 (6)	C426—H426	0.9500
			
Cl1···C314^i^	3.606 (5)	C414···H223^x^	3.0400
Cl1···H314^i^	2.8800	C415···H223^x^	2.8100
Cl1···H323^ii^	2.9900	C416···H223^x^	3.1000
Cl2···H115^iii^	3.0300	C425···H224^x^	2.8700
Cl2···H33	3.0000	C426···H224^x^	2.9000
P1···C23	2.765 (3)	C426···H43	2.8800
P1···P2	3.2360 (13)	H13···H116	2.2400
P2···P1	3.2360 (13)	H13···C116	2.9200
P2···C13	2.757 (3)	H23···C222	2.9600
P3···P4	3.1959 (13)	H23···H222	2.4700
P3···C43	2.752 (3)	H23···C126^iv^	3.0500
P4···C33	2.742 (3)	H33···H316	2.5100
P4···P3	3.1959 (13)	H33···C316	2.9900
P2···H322	3.0700	H33···Cl2	3.0000
C13···C23^iv^	3.563 (5)	H43···H416	2.4500
C23···C13^iv^	3.563 (5)	H43···C426	2.8800
C111···C422	3.563 (6)	H43···H426	2.2000
C112···C422	3.479 (7)	H113···C225^ii^	2.9900
C112···C421	3.449 (6)	H114···H125^xi^	2.5100
C13···H116	2.7000	H114···C224^ii^	3.0600
C113···C225^ii^	3.481 (6)	H115···Cl2^v^	3.0300
C13···H325^v^	3.0100	H116···H13	2.2400
C114···C224^ii^	3.548 (7)	H116···C13	2.7000
C116···C126	3.517 (6)	H116···H325^v^	2.5800
C23···H222	2.5900	H116···C214^iv^	3.0300
C126···C116	3.517 (6)	H122···C312	2.7500
C33···H316	2.7300	H122···H212	2.5200
C43···H426	2.7400	H122···C311	2.7400
C43···H416	2.5700	H125···H114^vii^	2.5100
C111···H126	2.8300	H126···C216^iv^	2.9900
C213···C325^vi^	3.545 (7)	H126···C116	2.8200
C214···C416^vi^	3.559 (8)	H126···C111	2.8300
C115···H425^vii^	3.0800	H212···C122	3.0600
C115···H326^v^	3.0500	H212···H122	2.5200
C116···H126	2.8200	H213···C323^vi^	3.0800
C116···H13	2.9200	H213···C324^vi^	2.9700
C216···C226	3.221 (6)	H214···H416^vi^	2.5200
C116···H325^v^	3.0900	H215···H425^viii^	2.5700
C221···C412	3.583 (6)	H216···C226	2.6300
C122···H315^i^	3.0000	H216···C221	2.7400
C122···H212	3.0600	H216···H226	2.4900
C123···H324^vi^	2.8600	H222···C23	2.5900
C123···H315^i^	2.8000	H222···H23	2.4700
C224···C114^viii^	3.548 (7)	H222···H324^v^	2.5100
C225···C113^viii^	3.481 (6)	H222···C324^v^	3.0700
C225···C413	3.543 (9)	H223···C415^x^	2.8100
C226···C412	3.561 (7)	H223···C416^x^	3.1000
C126···H23^iv^	3.0500	H223···C414^x^	3.0400
C226···C216	3.221 (6)	H224···C426^x^	2.9000
C211···H226	3.0700	H224···C425^x^	2.8700
C312···C326	3.454 (5)	H226···C211	3.0700
C314···Cl1^i^	3.606 (5)	H226···H322	2.5300
C214···H116^iv^	3.0300	H226···C216	2.9100
C214···H416^vi^	3.0700	H226···H216	2.4900
C315···C315^i^	3.588 (8)	H312···C321	2.8400
C216···H226	2.9100	H312···C414^vi^	3.0300
C216···H126^iv^	2.9900	H314···Cl1^i^	2.8800
C221···H216	2.7400	H315···C123^i^	2.8000
C221···H412	2.9100	H315···C122^i^	3.0000
C222···H23	2.9600	H316···C33	2.7300
C222···H412	2.9200	H316···H33	2.5100
C224···H413	3.0300	H322···H226	2.5300
C224···H114^viii^	3.0600	H322···P2	3.0700
C225···H413	3.0300	H323···Cl1^viii^	2.9900
C225···H113^viii^	2.9900	H324···H222^iii^	2.5100
C325···C213^ix^	3.545 (7)	H324···C123^ix^	2.8600
C326···C312	3.454 (5)	H325···C13^iii^	3.0100
C226···H216	2.6300	H325···H116^iii^	2.5800
C311···H326	2.9500	H325···C116^iii^	3.0900
C311···H122	2.7400	H326···C115^iii^	3.0500
C312···H122	2.7500	H326···C311	2.9500
C412···C226	3.561 (7)	H412···C221	2.9100
C412···C221	3.583 (6)	H412···C222	2.9200
C413···C225	3.543 (9)	H413···C224	3.0300
C314···H424^iii^	3.0300	H413···C225	3.0300
C416···C214^ix^	3.559 (8)	H414···C325^ix^	2.8700
C316···H33	2.9900	H414···C326^ix^	3.0100
C321···H312	2.8400	H416···H43	2.4500
C421···C112	3.449 (6)	H416···C43	2.5700
C422···C112	3.479 (7)	H416···H214^ix^	2.5200
C422···C111	3.563 (6)	H416···C214^ix^	3.0700
C323···H213^ix^	3.0800	H424···C314^v^	3.0300
C324···H222^iii^	3.0700	H425···H215^ii^	2.5700
C324···H213^ix^	2.9700	H425···C115^xi^	3.0800
C325···H414^vi^	2.8700	H426···H43	2.2000
C326···H414^vi^	3.0100	H426···C43	2.7400
C414···H312^ix^	3.0300		
			
P1—Cu1—P2	89.61 (4)	C125—C126—H126	120.00
P1—Cu1—P3	122.55 (4)	P3—C311—C312	118.2 (3)
P1—Cu1—P4	115.54 (4)	C312—C311—C316	118.7 (3)
P2—Cu1—P3	123.27 (4)	P3—C311—C316	122.7 (3)
P2—Cu1—P4	122.10 (4)	C311—C312—C313	120.3 (4)
P3—Cu1—P4	87.15 (4)	C213—C212—H212	119.00
Cl1—Cu2—Cl2	176.81 (7)	C211—C212—H212	120.00
Cu1—P1—C111	118.80 (11)	C212—C213—H213	120.00
Cu1—P1—C121	125.23 (11)	C312—C313—C314	120.4 (4)
C13—P1—C111	104.95 (15)	C214—C213—H213	120.00
C13—P1—C121	100.06 (15)	C313—C314—C315	120.0 (4)
C111—P1—C121	102.13 (15)	C213—C214—H214	120.00
Cu1—P1—C13	102.45 (11)	C215—C214—H214	120.00
Cu1—P2—C23	102.85 (11)	C314—C315—C316	120.7 (4)
Cu1—P2—C221	122.64 (11)	C216—C215—H215	120.00
C23—P2—C211	101.20 (16)	C214—C215—H215	120.00
C23—P2—C221	102.88 (15)	C215—C216—H216	120.00
C211—P2—C221	105.62 (17)	C211—C216—H216	120.00
Cu1—P2—C211	118.20 (15)	C311—C316—C315	119.9 (4)
Cu1—P3—C311	122.85 (11)	P3—C321—C326	121.3 (3)
Cu1—P3—C321	118.30 (11)	C322—C321—C326	118.9 (3)
Cu1—P3—C33	103.72 (11)	P3—C321—C322	119.7 (3)
C33—P3—C321	103.33 (15)	C321—C322—C323	121.1 (4)
C311—P3—C321	102.25 (15)	C221—C222—H222	120.00
C33—P3—C311	103.98 (15)	C223—C222—H222	119.00
Cu1—P4—C43	101.55 (11)	C322—C323—C324	119.2 (4)
Cu1—P4—C421	123.20 (12)	C222—C223—H223	120.00
C43—P4—C411	102.59 (18)	C224—C223—H223	120.00
Cu1—P4—C411	117.98 (15)	C225—C224—H224	120.00
C411—P4—C421	102.93 (17)	C223—C224—H224	120.00
C43—P4—C421	106.21 (19)	C323—C324—C325	120.6 (4)
P1—C13—C23	121.7 (3)	C226—C225—H225	120.00
P2—C23—C13	121.4 (2)	C224—C225—H225	120.00
P3—C33—C43	121.3 (2)	C324—C325—C326	120.0 (4)
P4—C43—C33	120.6 (3)	C225—C226—H226	120.00
P1—C111—C116	124.0 (3)	C321—C326—C325	120.2 (4)
C112—C111—C116	119.0 (3)	C221—C226—H226	120.00
P1—C111—C112	117.0 (3)	C412—C411—C416	118.9 (5)
C111—C112—C113	120.4 (4)	P4—C411—C412	118.8 (4)
C23—C13—H13	119.00	P4—C411—C416	122.2 (4)
C112—C113—C114	119.8 (4)	C313—C312—H312	120.00
P1—C13—H13	119.00	C411—C412—C413	120.3 (6)
C113—C114—C115	120.5 (4)	C311—C312—H312	120.00
C114—C115—C116	120.1 (4)	C314—C313—H313	120.00
C111—C116—C115	120.2 (4)	C312—C313—H313	120.00
P1—C121—C122	120.2 (3)	C412—C413—C414	120.5 (8)
P1—C121—C126	120.7 (3)	C313—C314—H314	120.00
C122—C121—C126	119.1 (4)	C413—C414—C415	119.7 (7)
C121—C122—C123	120.1 (4)	C315—C314—H314	120.00
C13—C23—H23	119.00	C316—C315—H315	120.00
P2—C23—H23	119.00	C414—C415—C416	121.0 (6)
C122—C123—C124	120.6 (4)	C314—C315—H315	120.00
C123—C124—C125	120.2 (4)	C315—C316—H316	120.00
C124—C125—C126	119.4 (4)	C311—C316—H316	120.00
C121—C126—C125	120.6 (4)	C411—C416—C415	119.6 (6)
C43—C33—H33	119.00	C422—C421—C426	118.7 (3)
P3—C33—H33	119.00	P4—C421—C422	117.6 (3)
P4—C43—H43	120.00	P4—C421—C426	123.7 (3)
C33—C43—H43	120.00	C421—C422—C423	120.9 (4)
P2—C211—C216	124.5 (3)	C321—C322—H322	120.00
P2—C211—C212	117.0 (3)	C323—C322—H322	119.00
C212—C211—C216	118.6 (3)	C422—C423—C424	120.0 (4)
C211—C212—C213	120.9 (4)	C324—C323—H323	120.00
C113—C112—H112	120.00	C322—C323—H323	120.00
C111—C112—H112	120.00	C423—C424—C425	120.3 (4)
C114—C113—H113	120.00	C325—C324—H324	120.00
C212—C213—C214	120.2 (5)	C323—C324—H324	120.00
C112—C113—H113	120.00	C424—C425—C426	119.7 (4)
C113—C114—H114	120.00	C326—C325—H325	120.00
C213—C214—C215	120.5 (5)	C324—C325—H325	120.00
C115—C114—H114	120.00	C421—C426—C425	120.5 (4)
C114—C115—H115	120.00	C325—C326—H326	120.00
C116—C115—H115	120.00	C321—C326—H326	120.00
C214—C215—C216	119.9 (4)	C411—C412—H412	120.00
C111—C116—H116	120.00	C413—C412—H412	120.00
C211—C216—C215	119.9 (4)	C414—C413—H413	120.00
C115—C116—H116	120.00	C412—C413—H413	120.00
P2—C221—C222	120.3 (3)	C413—C414—H414	120.00
C222—C221—C226	118.7 (3)	C415—C414—H414	120.00
P2—C221—C226	120.9 (3)	C416—C415—H415	120.00
C121—C122—H122	120.00	C414—C415—H415	119.00
C221—C222—C223	121.0 (3)	C411—C416—H416	120.00
C123—C122—H122	120.00	C415—C416—H416	120.00
C122—C123—H123	120.00	C423—C422—H422	120.00
C124—C123—H123	120.00	C421—C422—H422	119.00
C222—C223—C224	119.6 (4)	C422—C423—H423	120.00
C123—C124—H124	120.00	C424—C423—H423	120.00
C125—C124—H124	120.00	C425—C424—H424	120.00
C223—C224—C225	120.1 (4)	C423—C424—H424	120.00
C124—C125—H125	120.00	C424—C425—H425	120.00
C126—C125—H125	120.00	C426—C425—H425	120.00
C224—C225—C226	120.6 (4)	C421—C426—H426	120.00
C121—C126—H126	120.00	C425—C426—H426	120.00
C221—C226—C225	120.0 (4)		
			
P2—Cu1—P1—C13	11.99 (11)	Cu1—P4—C43—C33	−16.8 (3)
P2—Cu1—P1—C111	127.00 (12)	C411—P4—C43—C33	105.6 (3)
P2—Cu1—P1—C121	−100.03 (13)	C421—P4—C43—C33	−146.7 (3)
P3—Cu1—P1—C13	142.27 (11)	Cu1—P4—C411—C412	−57.5 (4)
P3—Cu1—P1—C111	−102.72 (12)	Cu1—P4—C411—C416	123.7 (4)
P3—Cu1—P1—C121	30.25 (14)	C43—P4—C411—C412	−168.1 (4)
P4—Cu1—P1—C13	−113.87 (11)	C43—P4—C411—C416	13.2 (4)
P4—Cu1—P1—C111	1.14 (13)	C421—P4—C411—C412	81.8 (4)
P4—Cu1—P1—C121	134.12 (13)	C421—P4—C411—C416	−97.0 (4)
P1—Cu1—P2—C23	−11.66 (11)	Cu1—P4—C421—C422	47.7 (4)
P1—Cu1—P2—C211	98.73 (13)	Cu1—P4—C421—C426	−133.1 (3)
P1—Cu1—P2—C221	−126.34 (14)	C43—P4—C421—C422	163.7 (3)
P3—Cu1—P2—C23	−141.38 (11)	C43—P4—C421—C426	−17.0 (4)
P3—Cu1—P2—C211	−31.00 (14)	C411—P4—C421—C422	−88.8 (3)
P3—Cu1—P2—C221	103.94 (14)	C411—P4—C421—C426	90.4 (4)
P4—Cu1—P2—C23	108.66 (11)	P1—C13—C23—P2	1.1 (4)
P4—Cu1—P2—C211	−140.96 (13)	P3—C33—C43—P4	0.8 (4)
P4—Cu1—P2—C221	−6.03 (14)	P1—C111—C112—C113	177.9 (4)
P1—Cu1—P3—C33	98.93 (12)	C116—C111—C112—C113	−1.0 (6)
P1—Cu1—P3—C311	−18.05 (14)	P1—C111—C116—C115	−177.0 (3)
P1—Cu1—P3—C321	−147.43 (12)	C112—C111—C116—C115	1.8 (6)
P2—Cu1—P3—C33	−146.92 (11)	C111—C112—C113—C114	0.1 (7)
P2—Cu1—P3—C311	96.10 (13)	C112—C113—C114—C115	0.0 (7)
P2—Cu1—P3—C321	−33.28 (14)	C113—C114—C115—C116	0.8 (7)
P4—Cu1—P3—C33	−19.78 (12)	C114—C115—C116—C111	−1.7 (7)
P4—Cu1—P3—C311	−136.76 (13)	P1—C121—C122—C123	178.6 (3)
P4—Cu1—P3—C321	93.86 (13)	C126—C121—C122—C123	−0.9 (6)
P1—Cu1—P4—C43	−105.01 (12)	P1—C121—C126—C125	−178.0 (3)
P1—Cu1—P4—C411	143.86 (16)	C122—C121—C126—C125	1.4 (6)
P1—Cu1—P4—C421	13.30 (18)	C121—C122—C123—C124	0.1 (7)
P2—Cu1—P4—C43	148.08 (12)	C122—C123—C124—C125	0.1 (7)
P2—Cu1—P4—C411	36.95 (17)	C123—C124—C125—C126	0.4 (7)
P2—Cu1—P4—C421	−93.61 (18)	C124—C125—C126—C121	−1.2 (7)
P3—Cu1—P4—C43	19.97 (12)	P2—C211—C212—C213	−179.3 (4)
P3—Cu1—P4—C411	−91.16 (16)	C216—C211—C212—C213	1.5 (6)
P3—Cu1—P4—C421	138.28 (18)	P2—C211—C216—C215	−179.4 (3)
Cu1—P1—C13—C23	−10.6 (3)	C212—C211—C216—C215	−0.1 (6)
C111—P1—C13—C23	−135.3 (3)	C211—C212—C213—C214	−2.2 (8)
C121—P1—C13—C23	119.2 (3)	C212—C213—C214—C215	1.6 (8)
Cu1—P1—C111—C112	52.6 (3)	C213—C214—C215—C216	−0.3 (8)
Cu1—P1—C111—C116	−128.7 (3)	C214—C215—C216—C211	−0.5 (7)
C13—P1—C111—C112	166.2 (3)	P2—C221—C222—C223	−177.3 (3)
C13—P1—C111—C116	−15.0 (4)	C226—C221—C222—C223	−2.6 (6)
C121—P1—C111—C112	−89.8 (3)	P2—C221—C226—C225	176.0 (3)
C121—P1—C111—C116	89.0 (3)	C222—C221—C226—C225	1.4 (6)
Cu1—P1—C121—C122	−0.7 (4)	C221—C222—C223—C224	2.5 (7)
Cu1—P1—C121—C126	178.8 (3)	C222—C223—C224—C225	−1.1 (7)
C13—P1—C121—C122	−113.8 (3)	C223—C224—C225—C226	−0.1 (7)
C13—P1—C121—C126	65.6 (3)	C224—C225—C226—C221	−0.1 (6)
C111—P1—C121—C122	138.4 (3)	P3—C311—C312—C313	172.9 (3)
C111—P1—C121—C126	−42.2 (3)	C316—C311—C312—C313	−0.1 (6)
Cu1—P2—C23—C13	9.1 (3)	P3—C311—C316—C315	−173.2 (3)
C211—P2—C23—C13	−113.6 (3)	C312—C311—C316—C315	−0.6 (6)
C221—P2—C23—C13	137.4 (3)	C311—C312—C313—C314	0.5 (6)
Cu1—P2—C211—C212	−29.1 (4)	C312—C313—C314—C315	−0.1 (7)
Cu1—P2—C211—C216	150.1 (3)	C313—C314—C315—C316	−0.6 (8)
C23—P2—C211—C212	82.2 (3)	C314—C315—C316—C311	1.0 (7)
C23—P2—C211—C216	−98.6 (4)	P3—C321—C322—C323	177.2 (3)
C221—P2—C211—C212	−170.9 (3)	C326—C321—C322—C323	−1.2 (6)
C221—P2—C211—C216	8.3 (4)	P3—C321—C326—C325	−177.1 (3)
Cu1—P2—C221—C222	88.3 (3)	C322—C321—C326—C325	1.3 (5)
Cu1—P2—C221—C226	−86.2 (3)	C321—C322—C323—C324	0.2 (7)
C23—P2—C221—C222	−26.4 (3)	C322—C323—C324—C325	0.8 (7)
C23—P2—C221—C226	159.1 (3)	C323—C324—C325—C326	−0.7 (7)
C211—P2—C221—C222	−132.1 (3)	C324—C325—C326—C321	−0.4 (6)
C211—P2—C221—C226	53.4 (3)	P4—C411—C412—C413	178.8 (5)
Cu1—P3—C33—C43	16.5 (3)	C416—C411—C412—C413	−2.4 (9)
C311—P3—C33—C43	146.0 (3)	P4—C411—C416—C415	−178.2 (4)
C321—P3—C33—C43	−107.5 (3)	C412—C411—C416—C415	3.1 (8)
Cu1—P3—C311—C312	−85.3 (3)	C411—C412—C413—C414	0.7 (11)
Cu1—P3—C311—C316	87.4 (3)	C412—C413—C414—C415	0.4 (11)
C33—P3—C311—C312	157.9 (3)	C413—C414—C415—C416	0.4 (9)
C33—P3—C311—C316	−29.5 (3)	C414—C415—C416—C411	−2.1 (9)
C321—P3—C311—C312	50.6 (3)	P4—C421—C422—C423	177.3 (4)
C321—P3—C311—C316	−136.7 (3)	C426—C421—C422—C423	−2.0 (6)
Cu1—P3—C321—C322	7.8 (4)	P4—C421—C426—C425	−177.9 (3)
Cu1—P3—C321—C326	−173.9 (2)	C422—C421—C426—C425	1.3 (6)
C33—P3—C321—C322	121.6 (3)	C421—C422—C423—C424	2.5 (7)
C33—P3—C321—C326	−60.1 (3)	C422—C423—C424—C425	−2.3 (7)
C311—P3—C321—C322	−130.6 (3)	C423—C424—C425—C426	1.6 (7)
C311—P3—C321—C326	47.7 (3)	C424—C425—C426—C421	−1.1 (7)

Symmetry codes: (i) −*x*+1, −*y*, −*z*+2; (ii) *x*−1/2, −*y*+1/2, *z*+1/2; (iii) *x*−1/2, −*y*+1/2, *z*−1/2; (iv) −*x*+2, −*y*, −*z*+2; (v) *x*+1/2, −*y*+1/2, *z*+1/2; (vi) −*x*+3/2, *y*−1/2, −*z*+3/2; (vii) −*x*+3/2, *y*−1/2, −*z*+5/2; (viii) *x*+1/2, −*y*+1/2, *z*−1/2; (ix) −*x*+3/2, *y*+1/2, −*z*+3/2; (x) −*x*+2, −*y*+1, −*z*+2; (xi) −*x*+3/2, *y*+1/2, −*z*+5/2.
